# Parasagittal Meningioma Mimicking Peroneal Neuropathy: A Case Report From Kurdistan, Iraq

**DOI:** 10.7759/cureus.96068

**Published:** 2025-11-04

**Authors:** Anjam Ibrahim Sulaiman Rowandizy, Ahmed A Al-Juboori

**Affiliations:** 1 Department of Surgery, Hawler Medical University, College of Medicine, Erbil, IRQ; 2 Department of Neurosurgery, Dr. Sa’ad AL-Witri Hospital for Neurosciences, Baghdad, IRQ

**Keywords:** central cause, iraq, isolated foot drop, motor cortex compression, parasagittal meningioma

## Abstract

This case report describes a rare presentation of isolated right foot drop due to a left parasagittal meningioma compressing the motor cortex. A 69-year-old man experienced progressive motor deficits without accompanying sensory loss or typical upper motor neuron (UMN) signs, leading to an initial misdiagnosis of peripheral neuropathy. Brain MRI revealed a left parasagittal extra-axial lesion consistent with a meningioma. Surgical resection confirmed a meningothelial meningioma, and the patient showed significant motor improvement postoperatively. This case emphasizes the importance of considering central causes in atypical presentations of foot drop and advocates for early neuroimaging to avoid diagnostic delays.

## Introduction

The parasagittal region is considered the second most common origin of meningioma, arising from the dura mater along the convex hemispheric walls and accounting for approximately 20%-30% of all intracranial meningiomas [1]. Surgical resection remains the primary treatment modality, although it carries the risk of various postoperative complications, including wound infection, cerebrospinal fluid leak, hydrocephalus, hematoma, venous infarction, cerebral edema, and air embolism [2, 3].

Although foot drop is commonly associated with peripheral nerve pathologies, lesions of the central nervous system may also result in foot drop. Such central causes include mass lesions in the interhemispheric motor cortex, lacunar infarctions of the internal capsule or cerebral peduncles, demyelinating diseases, or spinal cord pathologies. However, in most of these cases, foot drop is typically accompanied by additional neurologic deficits on examination [4, 5]. Isolated foot drop as the sole manifestation of a cortical lesion is exceedingly rare. This atypical presentation often leads to misdiagnosis, as the absence of upper motor neuron (UMN) signs results in initial misattribution. Furthermore, electrodiagnostic studies such as nerve conduction and electromyography can be misleading, as misinterpretation of F-wave responses and motor unit recruitment patterns may obscure the central origin of the deficit. These factors contribute to delays in accurate diagnosis and timely management of central foot drop caused by parasagittal meningioma [6].

This case report presents a rare instance of isolated right foot drop resulting from a parasagittal meningioma compressing the motor cortex of the right hemisphere.

## Case presentation

A 69-year-old right-handed man from Kurdistan, Iraq, presented with a three-month history of progressive right-sided foot drop, manifesting as difficulty with dorsiflexion and frequent tripping. The onset was insidious and gradually worsened, interfering with ambulation and daily activities. Because there was no back pain, paresthesia, or sensory disturbance, the initial clinical impression was peroneal neuropathy. The patient denied preceding trauma, constitutional symptoms, diabetes mellitus, or previous neurosurgical intervention.

On neurological examination, there was an isolated motor deficit affecting the right lower limb. Ankle dorsiflexion was graded 2/5 on the Medical Research Council (MRC) scale [7], while ankle eversion and inversion were both preserved at 5/5. Knee extension (L3-L4) and hip flexion (L2-L3) were full-strength (5/5), excluding a multi-root or peripheral pattern. No visible muscle wasting or fasciculations were observed, and calf circumferences were symmetrical (right 34.5 cm vs left 34.6 cm), providing objective evidence against chronic denervation. Muscle tone was increased distally in a spastic distribution, and deep tendon reflexes were brisk in the right lower limb, with a preserved and symmetrical Achilles reflex. The right plantar response was extensor, confirming a UMN pattern. There was no sensory deficit in the peroneal nerve distribution, no Tinel sign at the fibular neck, and no paraspinal tenderness. Bowel and bladder functions were intact, and the contralateral limb and both upper extremities were normal.

**Figure 1:**
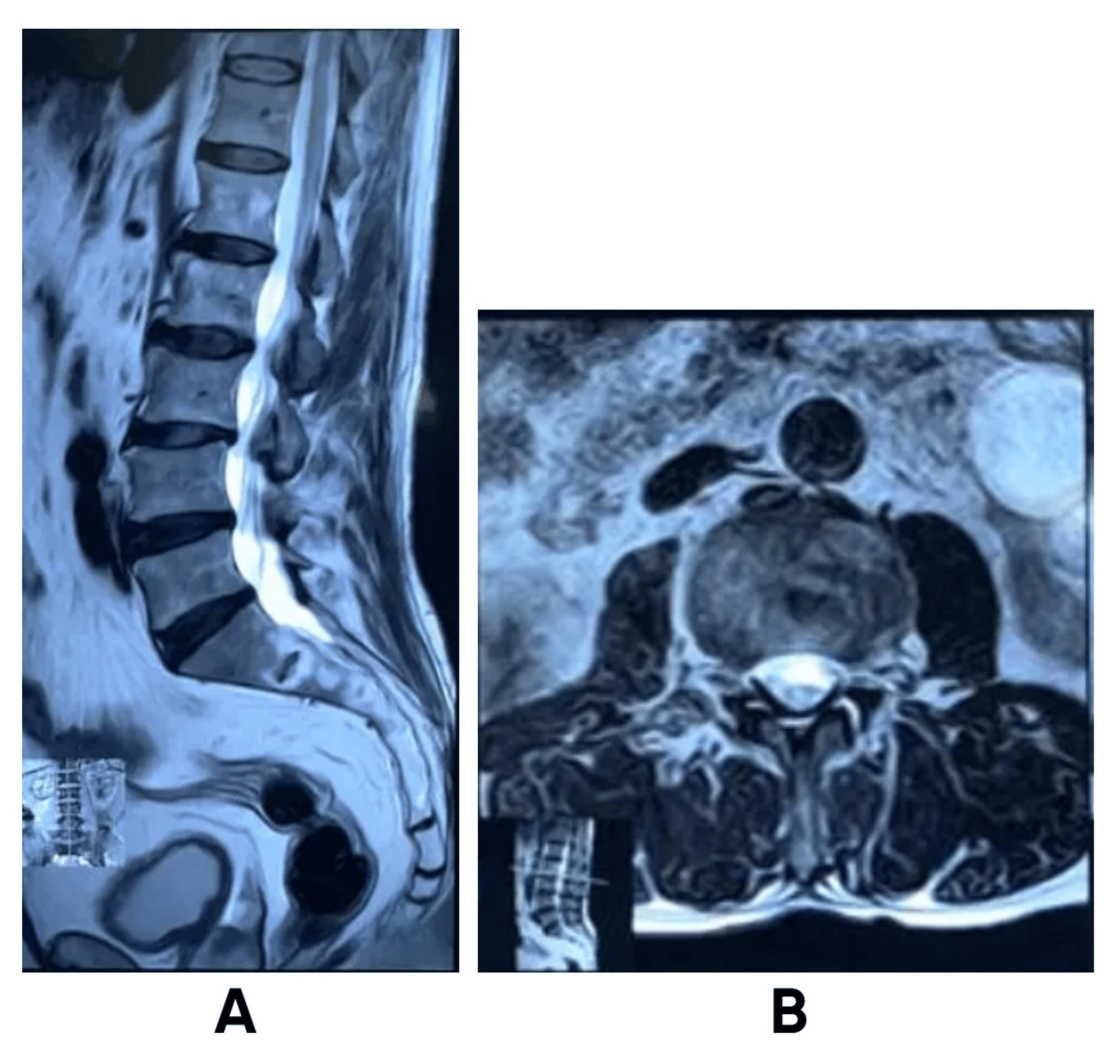
Preoperative lumbosacral spine MRI findings (A) Sagittal T2-weighted image showing preserved vertebral alignment with mild disc protrusions and no evidence of compressive or intradural pathology. (B) Axial T2-weighted image at the L4–L5 level demonstrating intact neural structures and absence of nerve root compression.

Lumbosacral MRI was performed and revealed unremarkable findings, with preserved vertebral alignment, intact intervertebral discs, and no evidence of compressive or intradural pathology, thereby excluding a spinal cause for the patient’s foot drop (Figure 1).

**Figure 2:**
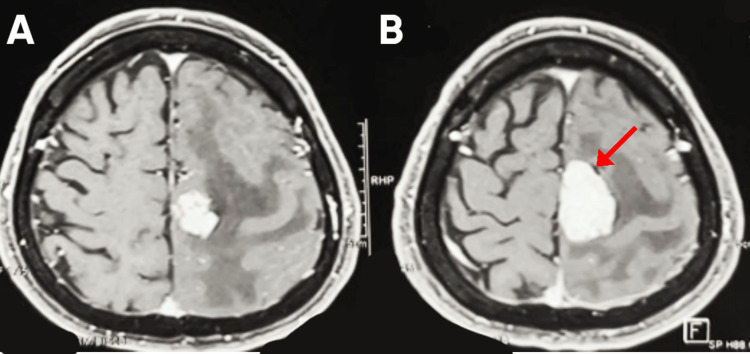
Preoperative axial post-contrast T1-weighted MRI of the brain (A) Axial image showing a left parasagittal dural-based lesion with homogeneous enhancement and associated mass effect. (B) Axial image demonstrating the same lesion (red arrow) compressing the adjacent motor cortex, consistent with a parasagittal meningioma.

**Figure 3:**
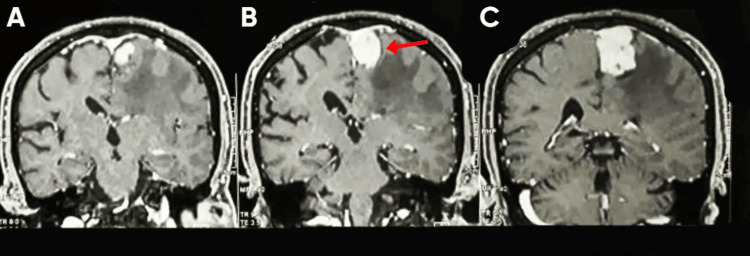
Preoperative coronal post-contrast T1-weighted MRI of the brain (A–C) Sequential coronal sections demonstrating a left parasagittal dural-based enhancing lesion. (B) The red arrow indicates the homogeneously enhancing meningioma compressing the adjacent motor cortex, consistent with a parasagittal meningioma.

A preoperative brain MRI with contrast demonstrated a left parasagittal extra-axial enhancing mass consistent with meningioma. The lesion appeared dural-based, showing homogeneous enhancement with associated mass effect on the adjacent motor cortex. Perilesional edema was evident (Figures 2, 3).

**Figure 4:**
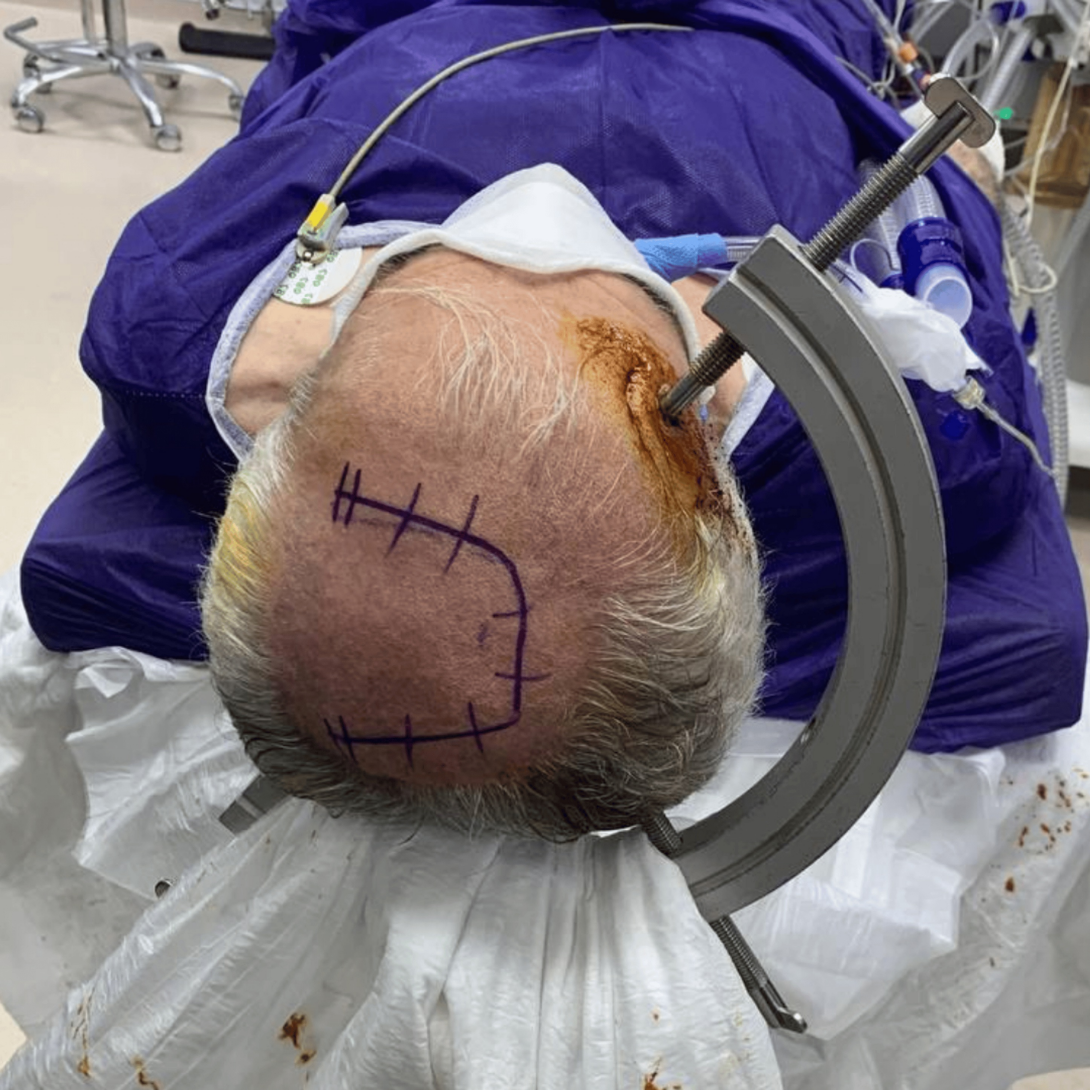
Intraoperative photograph showing the patient’s head fixed in a six-pin head fixation (Sugita) device. The planned left parasagittal curvilinear incision is marked on the scalp to provide optimal exposure for tumor resection.

The patient was positioned supine with the head secured in a Sugita head holder (Mizuho Corporation, Tokyo, Japan) to ensure rigid fixation. A left parasagittal curvilinear skin incision was planned, guided by craniometrics to identify surface landmarks, to provide adequate exposure of the tumor site while preserving venous structures. Standard aseptic preparation and draping were performed prior to craniotomy (Figure 4).

**Figure 5:**
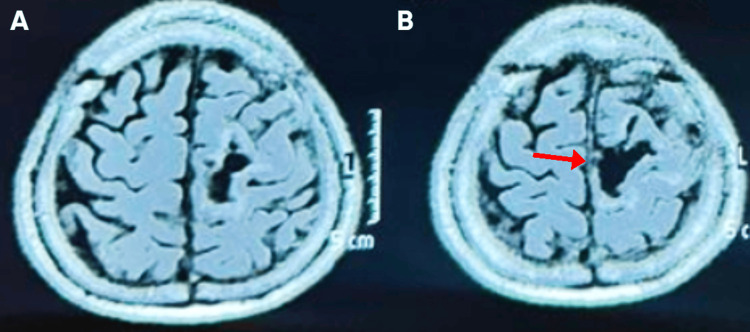
Postoperative axial MRI images of the brain Figures A and B show interval changes at the left parasagittal surgical site following tumor resection. The resection cavity is visible (arrow in B), with no residual enhancing mass or significant mass effect identified, consistent with gross total excision.

**Figure 6:**
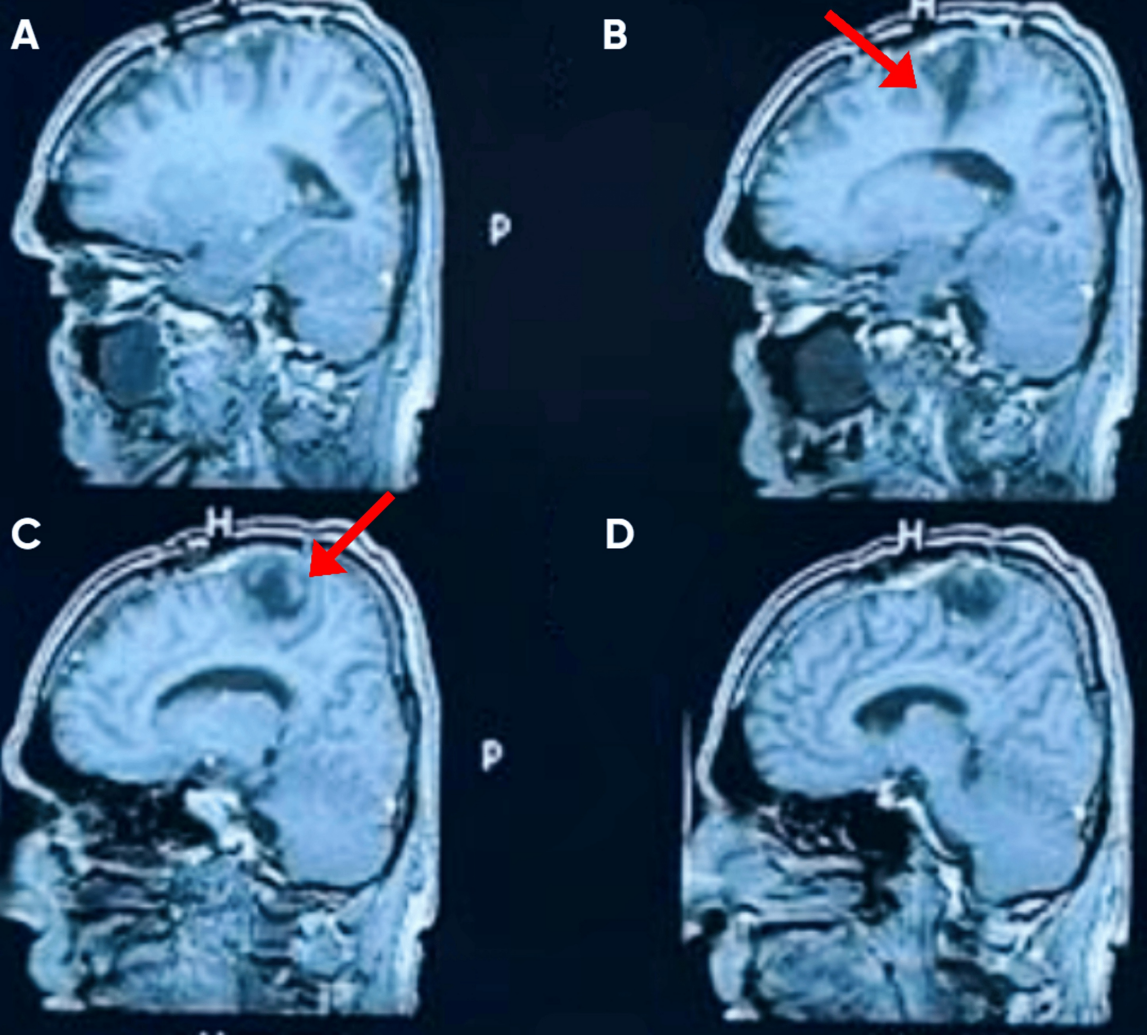
Postoperative sagittal post-contrast T1-weighted MRI images of the brain Figures A–D show interval changes at the left parasagittal surgical site following tumor resection. Arrows in Figures B and C indicate the postoperative cavity and adjacent dural enhancement (empty bed).

Intraoperatively, the tumor was noted to be firm, vascular, and adherent to the dura and underlying cortex, consistent with meningioma. The mass was totally resected. Histopathological examination confirmed a meningothelial meningioma. Postoperatively, the patient demonstrated early signs of motor recovery, with improved voluntary dorsiflexion over the subsequent weeks. Postoperative axial MRI demonstrated interval resection of the left parasagittal mass with postoperative changes at the surgical site. No residual enhancing lesion, significant mass effect, or midline shift was observed, indicating gross total resection and favorable early postoperative findings (Figures 5-6). At the three-month follow-up, motor strength improved to 4/5 on the MRC scale [7], with no recurrence noted on follow-up imaging.

## Discussion

Foot drop is typically attributed to peripheral nervous system pathology, with common etiologies including peroneal neuropathy and lumbosacral radiculopathy. Central causes, such as lesions of the motor cortex, are far less common and often overlooked. Isolated foot drop due to parasagittal meningioma represents an infrequent manifestation of a supratentorial lesion [4, 5].

In our case, a left parasagittal meningioma caused isolated right-sided foot drop without initial UMN signs, mirroring diagnostic challenges observed in other reports. Notably, Bilić et al. (2019) [8] described a 79-year-old woman with isolated foot drop due to a parasagittal meningioma, who initially had no UMN signs. Central involvement was only suspected after normal neurophysiological studies and evolving reflex asymmetry, prompting brain imaging and subsequent tumor resection. Similarly, Narenthiran et al. (2011) [5] presented a 78-year-old woman with progressive foot drop over five years, eventually attributed to a parasagittal meningioma. Despite the chronicity of symptoms, initial examinations lacked definitive UMN signs, leading to delayed diagnosis and unnecessary spinal investigations. Brain imaging revealed the lesion, but the patient declined surgery.

A broad differential diagnosis must be considered when evaluating isolated foot drop. The most frequent etiologies are peripheral in origin, such as common peroneal neuropathy, L5 radiculopathy, or plexopathy, which typically present with sensory disturbances, neuropathic pain, or a history of trauma or compression [9]. Systemic conditions such as diabetes mellitus may also predispose to peripheral neuropathy, often producing a mixed sensory-motor deficit rather than a purely motor presentation [10]. Motor neuron disease and anterior horn cell pathologies may cause progressive weakness, but these are usually bilateral and accompanied by muscle wasting or fasciculations, making them less likely in an isolated unilateral presentation [11].

Central causes, though less common, are diagnostically important. These include ischemic lesions, such as lacunar infarcts involving the internal capsule or cerebral peduncles, as well as demyelinating diseases like multiple sclerosis and space-occupying lesions, including parasagittal meningiomas or gliomas that involve the motor cortex [12]. Recognizing this spectrum of differentials underscores the need for careful neurological examination and early neuroimaging to distinguish central from peripheral etiologies and avoid misdirected investigations.

These cases, along with our own, emphasize a critical diagnostic gap: central causes of isolated foot drop may not present with classic UMN signs and are frequently misattributed to peripheral pathology. Neuroimaging is often only pursued after standard evaluations fail to explain the symptoms. This delay in recognition can postpone potentially curative neurosurgical intervention.

## Conclusions

This case highlights that parasagittal meningiomas may present solely with isolated foot drop, leading to potential misdiagnosis as peripheral neuropathy. Clinicians should maintain a high index of suspicion for central causes when peripheral investigations are inconclusive. Early neuroimaging and multidisciplinary evaluation are crucial to ensure timely surgical intervention and favorable neurological recovery.
